# Supplementary material for: Are carbon emissions trading and green financial instruments synergistic? -Comprehensive quantitative research based on content analysis
Source: PLoS One. 2024 Mar 7;19(3):e0298601. doi: 10.1371/journal.pone.0298601 (PMC10919601; doi:10.1371/journal.pone.0298601)
Supplement: S1 Data — (ZIP) [file pone.0298601.s001.zip › Data/地级市Z_F工作报告.zip!/╣≤╓▌/╣≤╤⌠╩╨/└┤╫╘╣1⁄2╓┌║┼í╛┬φ┐╦╩2╛▌═°í┐_2013.docx]

各位代表：

　　现在，我代表市人民政府，向大会作政府工作报告，请予审议，并请市政协委员、列席人员提出意见和建议。

　　一、2012年工作回顾

　　过去一年，我们在省委、省政府和市委的正确领导下，在市人大、市政协的监督支持下，认真学习贯彻党的十八大精神，抢抓国发〔2012〕2号文件历史性政策机遇，紧扣“主基调”、实施“主战略”，在“坚持走科学发展路，加快建生态文明市”的进程中，经济社会呈现出发展加速、质量提升、环境优化、民生改善、社会稳定、创新突破的良好局面，为实现“一二一”奋斗目标奠定了更加扎实的基础。

　　——2012年，是经济稳中快进之年。全市多项经济指标增速继续保持在全国省会城市前列。全年实现生产总值1700.3亿元，实际增长17%，对全省经济增长贡献率达28.8%；完成全社会固定资产投资2483亿元、增长55%；规模以上工业增加值480亿元、增长22%；社会消费品零售总额683亿元、增长16.9%；财政总收入488亿元、增长21.6%，公共财政预算收入241.2亿元、增长28.9%，分别比上年净增加87亿元和54亿元。金融机构存、贷款余额分别达4394.4亿元、3479.5亿元，增长21.9%、15.4%；银行表外融资规模达1160亿元,占全省的60%。

　　——2012年，是结构优化调整之年。着力做特一产、做强二产、做优三产，三次产业结构优化为4.2：42.2：53.6。从产业内部结构看，一产中粮经比调整为3:7,畜牧业产值占农业总产值比重达30%以上；二产中轻重工业比从45:55调整为47:53，高新技术工业增加值98.4亿元、占规模以上工业增加值的20.5%；三产中现代服务业实现增加值428亿元，占服务业增加值的47%。从投资结构看，全社会固定资产投资中，产业投资占比37.7%、基础投资占比19.4%、民生及公共投资占比42.9%。从税收结构看，三次产业税收贡献比为0.2：45.9：53.9。从所有制经济结构看，民营经济实现增加值810亿元，占全市生产总值的47.6%，提高了3.6个百分点。

　　——2012年，是改革创新突破之年。深入推进城市基层管理体制改革，撤销街道办事处，建立社区服务中心，把政府公共服务送到群众身边，被列为全国从整体上加强和创新社会管理三个大城市试点之一。经国务院批准，设立观山湖区和新花溪区。在全国首家组建市生态文明建设委员会、区（市、县）生态文明建设局，国家批准实施《贵阳建设全国生态文明示范城市规划》。教育、文化、医药卫生体制改革深入推进，贵阳幼儿师范学校升格为高等专科学校，我市被评为全国文化体制改革先进地区，列为全国全科医生执业方式和服务模式改革试点城市。高新区被列为国家级新型工业化产业示范基地、科技兴贸创新基地；经开区被列为全国电子商务示范基地。

　　——2012年，是开放水平提高之年。生态文明贵阳会议、酒博会、国际国内体育赛事等重要对外交流活动成功举办，规模及成效再创新高。完成贵阳综合保税区规划编制并上报国家审批，启动无水港建设。朗玛公司成功上市，实现了贵州创业板上市公司零的突破。新开通国际国内直航航线17条，全市入境游客达11.5万人（次），增长110%，贵阳机场获落地签证权。开放型经济迈上新台阶，引进内资到位资金1354.5亿元、增长95.9%；实际利用外资4.7亿美元、增长70%；外贸进出口总额达50.5亿美元、增长34%，获批国家外贸转型升级示范基地，被评为中国十大最具潜力会展城市、福布斯·中国大陆最佳商业城市。

　　——2012年，是城乡发展提升之年。国务院批准新一轮《贵阳市土地利用总体规划》，中心城区建设用地规模从155.3平方公里调增至341.3平方公里；完成“三区五城五带”总体规划，实现城乡规划全覆盖。富源南路、桐荫路、东站路、盐沙路建成通车，“三环十六射”骨干路网形成闭合“内循环”；龙洞堡机场扩建（二期）、市域快铁、轨道交通、跨区域高速公路建设加快推进，金清线建成通车，“外循环”城市交通体系逐步完善。启动二环四路城市带15个功能板块建设和1560万平方米棚户区城中村改造。城乡规划展览馆、孔学堂及花溪十里河滩国家城市湿地公园、小车河湿地公园等精品工程建成投用，红枫湖——百花湖成功申报为国家湖泊保护试点和国家级城市湿地公园，国际金融中心、多彩贵州城等一批功能性设施顺利推进，南明河流域综合治理大规模、系统性实施。农村生产生活条件不断改善。实施村庄整治50个，完成农房整治2200户;建成农村公路220公里；开工建设7座水源工程，完成17座病险水库除险加固、4条中小河流治理和376处涉及23.9万人的农村人饮安全项目；投资5.6亿元实施农村电网改造升级和县级电网工程建设；新增耕地300公顷；完成营造林23.7万亩，石漠化治理132.8平方公里，森林覆盖率达43.2%。荣获CCTV2012年度中国城市品牌奖，“爽爽的贵阳”对外形象大幅提升！

　　——2012年，是民生持续改善之年。民生支出占公共财政预算支出的60%。其中，投入资金24亿元，圆满完成“十件民生实事”。社会保障体系不断完善，城镇新增就业17.2万人，城镇登记失业率控制在3%以内；城乡低保标准分别提高11.4%、12.5%，累计发放城乡低保金3.7亿元，惠及13.2万人；城镇职工基本养老、医疗、失业、工伤、生育保险征缴扩面，城乡居民社会养老保险制度全覆盖，60岁以上城乡居民实现应保尽保，向80岁以上老人发放长寿补贴4535万元。61.3万流动人口办理“城市绿卡”，享受同城化服务。新开工保障性住房27.6万平方米，竣工16万平方米，共有2064户住房困难群体入住新居。对32万贫困人口实施扶贫攻坚，13个贫困乡镇减贫“摘帽”，实现整市脱贫。全市人均生产总值达6090美元，城市居民人均可支配收入、农民人均纯收入分别为22525元、8710元，增长16%、18%，人民生活水平逐年提高！

　　——2012年，是社会全面进步之年。加大社会治安综合治理力度，刑事立案数下降2.1%，破案数上升9.1%，抓捕数上升19.5%,市民安全感上升5个点，达到89.37。安全生产事故起数、死亡人数分别下降21.1%、25.2%。居民消费价格涨幅控制在3%以内，物价保持总体稳定。中心城区中学学位数、学前教育学位数分别增加3794个、6052个，小学、初中辍学率分别为2%、2.9%，均控制在省下达目标范围内；花溪大学城、清镇职教城加快建设并有序入驻，启动建设百花湖国际学校。扎实推进医药卫生改革，药品价格下降12%、住院费用下降11%。计生、科技、体育等事业积极推进，国防、国家安全、司法行政、民族宗教、移民、史志、档案、外事、侨务、对台、气象、老龄、关心下一代、慈善、残疾人、红十字、爱国卫生、工青妇等工作全面发展。

　　——2012年，是政府建设提质之年。坚持建设“创新型、服务型、法治型、廉洁型”政府，推动政府工作提速提质提效。牢记宗旨，为民执政。始终把群众呼声作为“第一信号”，在想问题、作决策、办事情中广泛问需于民、问政于民、问计于民。建立联系群众新机制，深入开展领导干部挂帮企业、联系村寨、“访民情”、“摆家常”等活动。开拓创新，高效理政。深入开展作风效能建设，进一步改进工作作风、缩减审批环节，优化工作规程，改善服务环境。通过“百姓——书记市长交流台”、贵阳电视台“执行力”栏目等载体，打造高效办事直通车，推动政府效能提升。被评为中国城市服务型政府公众视角十佳城市。健全机制，依法行政。主动接受人大、政协监督，加强与各民主党派、工商联、社会团体和无党派人士的联系沟通。办理人大代表建议165件，政协提案350件，办结率和满意率均为100%。提请市人大常委会审议地方性法规（草案）3件，制定政府规章20件，受理行政复议630件。完善制度，廉洁从政。切实加强惩治和预防腐败体系建设，加大行政监察和审计监督力度，建立公共资源阳光交易中心，全面推进廉政风险防控管理，强化对公共权力运行的监督和制约，坚决纠正部门和行业不正之风。对291个项目实施前置审计，核减金额32亿元；严肃查处违纪违法案件209件，给予党政纪处分243人。政府廉政建设和反腐败工作取得新实效！

　　回顾一年来的发展，我们深切地感到：一次次智慧的碰撞，换来一个个创新和突破；一滴滴汗水的抛洒，推动一步步发展和变化。成绩源于齐心之努力，成功源于共同的坚持！在此，我代表市人民政府，向辛勤工作在全市各条战线、各行各业的广大工人、农民、知识分子、干部、市民群众，向尽心履职的人大代表、政协委员，向驻筑人民解放军指战员、武警官兵、公安民警、中央（省）驻筑单位，向所有关心支持贵阳发展的各民主党派、工商联、无党派人士、人民团体和社会各界人士，致以崇高的敬意和衷心的感谢！

　　总结一年来的工作，我们清醒地看到：我市在经济社会发展中仍然存在投资转化率不高，产业结构不优，自主创新能力不强，改善民生任务较重，工作效能还需进一步提高等问题。对此，我们将找准原因、着力破题、切实解决。

　　二、2013年工作安排

　　新的一年站在新的起点，孕育新的梦想！今年，是全面贯彻落实党的十八大精神的开局之年、是深入实施“十二五”规划承上启下之年、是迈向生态文明新时代的大干之年。做好今年各项工作，意义重大，使命光荣！

　　今年政府工作的总体要求是：坚持以邓小平理论、“三个代表”重要思想、科学发展观为指导，全面贯彻落实党的十八大精神，在省委、省政府和市委的正确领导下，抢抓新机遇，迎接新挑战，把握“主基调”，实施“主战略”，坚持“十破十立”，“创新突破，好中快进”，在建设全国生态文明示范城市中“作表率、走前列”，为到2015年在全省率先建成全面小康社会奠定坚实基础。

　　今年经济社会发展的主要预期目标是：生产总值增长17%；财政总收入增长15%，公共财政预算收入增长17%；全社会固定资产投资增长30%；社会消费品零售总额增长18%；实际直接利用外资增长30%，外贸进出口总额增长20%；工业经济比重提高到32.7%，民营经济比重提高到50%以上，信息化发展指数提高到0.8；城市居民人均可支配收入增长17%，农村居民人均可支配收入增长18%；城镇登记失业率控制在4%以内；城镇化率提高2个百分点；森林覆盖率提高1.5个百分点；人口自然增长率控制在5％。以内；居民消费价格指数控制在省调控目标范围内；完成省下达的节能减排任务；群众安全感和市民幸福指数进一步提升。

　　今年经济社会发展的工作重点是：牢牢把握“坚持走科学发展路，加快建生态文明市”的总路径、“创新突破，好中快进”的总原则、“四化同步”的总抓手和“提前五年全面小康”的总目标，紧紧抓住政策叠加的最大机遇、投资拉动的最大动力、城镇发展的最大内需和改革开放的最大红利，紧扣全省“5个100工程”，突出“六个更加注重”。

　　（一）更加注重生态文明建设，致力推进绿色低碳发展

　　生态文明是人类文明发展的潮流和趋势，是实现全面、协调、可持续发展的必然要求。按照市委九届二次全会“迈向生态文明新时代”的决定，紧扣“五位一体”总布局，切实贯彻落实《关于实施<贵阳建设全国生态文明示范城市规划>的意见》，系统谋划、分类指导、试点先行、重点突破，为建设“绿色经济崛起、幸福指数更高、城乡环境宜人、生态文化普及、生态文明制度完善”的全国生态文明示范城市，打造天蓝、地绿、水清、人爽的宜居新家园夯实基础。

　　一是坚持消费低碳化、环境人文化，广泛普及生态理念。实施低碳建筑工程，大力推广绿色建筑,完成272万平方米可再生能源建筑应用示范任务，创建“全国公共建筑节能改造重点示范城市”，推进建筑发展绿色转型。实施低碳交通工程，加大新能源汽车的推广应用，加快建设公共交通、轨道交通、慢行交通相配套的出行系统，创建“公交都市”，大力提高公交出行分担率，打造顺畅交通、绿色出行。实施低碳生活工程，弘扬生态文明价值观，繁荣生态文化，提高全社会的生态文明意识，倡导低碳生活理念和低碳消费方式，积极创建生态文明社区、企业、学校、单位和家庭，努力形成人人参与、自觉践行、模范遵守的生态文化氛围，让生态文明理念深刻植入城市发展和市民生活之中。

　　二是坚持生产清洁化、利用高效化，切实保护生态环境。积极顺应自然规律、尊重经济规律，促进环境换增长向环境促增长转变。把循环经济理念贯穿到园区发展、企业生产的各个环节，提高资源就地转化率和废物再利用率，实现资源深度开发和循环利用。进一步健全节能减排目标责任制，公开发布PM2.5监测数据和自来水水质检测数据，加快环保技术服务、排污权交易、合同能源管理、再生资源利用、重点污染源在线监控“五大体系”建设，加快淘汰落后产能，深入治理农村面源污染，整治关停一批污染企业，努力实现经济持续增长、污染持续下降、生态持续改善。

　　三是坚持生态产业化、产业生态化，着力提高生态效益。始终围绕生态价值、经济价值、社会价值“三个最大化”目标，构建可持续的生态效益价值体系。大力实施退耕还林还草、天然林和环城林带保护等林业生态经济建设，在实施二环四路、环城高速公路景观改造中，规模化发展高附加值的生态农业，建设环城绿色产业经济带，实现美化城市环境与增加群众收入的有效统一。从严保护“五口水缸”和饮用水源，科学化、系统性实施南明河综合整治，精心守护好城市“生命之源”，保护好“母亲河”！改造提升花溪十里河滩国家城市湿地公园和黔灵山公园，加快推进红枫湖——百花湖国家湿地公园、小车河湿地公园（二期）和小关湖城市湿地公园规划建设，进一步修复水——林——湿地城市生态系统，打造城市“绿色净化器”。大力实施石漠化和水土流失治理，加大城市三环内采石迹地修复，充分利用低丘缓坡规划建设白云金山等山体公园，十二滩、乐湾国际等山地户外体育旅游休闲基地。通过质量效益型生态发展，把山建成城市发展的生态屏障、群众致富的“绿色银行”！

　　（二）更加注重扩大内需，拉动经济快速发展

　　坚持速度与质量、规模与效益并重的要求，继续把扩投资、促消费作为扩大内需的两大抓手，加快形成新的增长方式，确保有效益的投资、无水分的增长、可持续的发展。

　　一是继续扩大有效投资，保持投资合理增长。着力抓好重大项目投资。围绕全年实现3000亿元投资目标，新开工建设天河潭新城、贵州复烤厂异地技改搬迁、世纪恒通信息产业中心和息烽煤电磷、煤电化一体化循环经济基地等57个项目，加快建设皓天光电LED蓝宝石、振华新材料新能源产业基地、首钢贵阳特殊钢新材料基地、奇瑞配套产业园等70个续建项目，建成拜特制药生产基地、黔轮胎异地搬迁、申发钢结构加工基地等一批项目，确保170个省级重大工程和重点项目顺利推进并力争完成投资1000亿元。着力优化投资结构。围绕产业投资达40%以上、民间投资达50%以上的目标，着力优化投资结构，努力提高投资强度和投资效益系数，让今天的投资成为明天的产业。年内确保新开工建设千万元以上工业投资项目100个以上，建成投产千万元以上工业投资项目100个以上。着力强化要素保障。进一步创新工作方式，有效解决好融资、供地、以及水、电、油、运、气、信息网络、征收安置、行政审批等问题，做实项目前期工作，强化督促检查，抓好要素保障，促进项目有序快速推进。

　　二是有效扩大消费需求，激发内需潜能。科学把握省会城市的消费需求结构和辐射消费空间，充分发挥消费对经济增长的拉动作用，确保社会消费品零售总额达800亿元以上。大力增强居民消费能力。通过扩大就业、完善职工工资正常增长机制、落实好收入分配调节政策等措施进一步提高居民收入，通过优化信贷政策、优惠服务业税费等措施培育消费热点，积极提升居民收入性、信贷性、预期性三大消费能力。着力丰富消费业态。扩大优质教育、文化、医疗等服务型消费，积极培育和提升旅游、休闲、健身、娱乐、住房、汽车等消费热点，大力发展电子商务、电视购物、租赁消费等新兴消费业态，继续开展送家电、农机具下乡等活动，推动消费差异化、多元化发展，全面增强省会城市的区域消费吸引力和辐射力。积极改善消费环境。加快大型商业综合体和特色商业街区建设，进一步推进社区平价生鲜商店、平价猪肉直销店、物流配送体系、农村现代流通网络等消费平台建设，着力完善诚信消费、质量安全、消费者权益保护体系，营造良好的消费环境，释放消费潜能。

　　（三）更加注重产业升级，构建现代产业体系

　　一是大力推动工业升级。始终坚持把优势资源和优势力量投向工业，推进“三大升级计划”，实现工业总量大跨越、质量大提升，确保规模以上工业总产值和增加值分别增长21%以上。实施工业结构升级计划。加大传统产业的改造和战略性新兴产业的培育，选准突破点，通过科技创新、资源整合、加大投入、集群发展，加快把材料产业、装备制造业、信息产业、医药食品（健康）产业等四大优势产业优先培育成千亿级产业，力争四大产业新增产值260亿元以上。实施企业规模升级计划。通过细化本地企业横向配套、支持本地产品采购、规模以上生产企业成立销售公司、加大技术改造、优化产品结构、强化品牌建设等措施，大力实施“百亿企业培育工程”、“中小企业成长工程”、“微小企业发展工程”，促进广铝、益佰、华能焦化等一大批规上企业扩大产能，打造一批骨干龙头企业和中小型实体企业。对招商引资项目进行系统梳理，千方百计促进投产达产，做大存量、扩大增量，力争新增规模以上企业40户，产值超20亿元企业3户、10亿元企业10户，培育3户企业上市，壮大“经济细胞”。实施园区水平升级计划。按照“用地集约、布局集中、企业集聚、产业集群”的原则和园区与园区之间、园区内企业之间相互配套的要求，加快水、电、路、气、信息网络为重点的设施建设，建成标准厂房38万平方米，完成园区基础设施投资290亿元；围绕提高项目入园率、落地率、开工率和投产率，完成园区产业投资490亿元以上，园区工业总产值达1400亿元。高新区、经开区要全力夯实基础、发挥优势，促进高新技术产业创新发展、跨越提升！

　　二是大力推动服务业升级。发挥省会城市优势，切实把服务业作为扩大就业的最大容纳器、结构调整的最佳着力点、三产提速的最大支撑力，坚持生产性服务业与生活性服务业、传统服务业和现代服务业并重发展。打造金融、物流、商贸“三大区域性中心”。围绕打造区域性金融中心，继续实施“引金入筑”工程，新增2至4家银行在筑设立分支机构；力争村镇银行在区（市、县）实现全覆盖，支持贵阳银行加快发展，推进农商行发展壮大；加快国际金融中心建设，激活城市金融要素。围绕打造区域性物流中心，抓好流通领域物流示范城市建设，推进跨区域高速公路、铁路建设，重点建设龙洞堡国际航空物流园和扎佐、清镇、金华三大区域性物流园，大力发展第三方物流。围绕打造区域性商贸中心，重点推进花溪农产品物流园（二期）、西南国际商贸城、孟关汽贸城（二期）等建设，规划布局中药材、茶叶、酒类等特色专业市场，加快老城区小商品、服装、建材、家具等交易市场搬迁改造,优化功能布局，促进市场集聚化、规模化、专业化发展。提升“两大品牌”。围绕提升“爽爽贵阳·避暑之都”旅游品牌知名度，推进旅游标准化、国际化建设，优化提升花溪十里河滩、小车河城市湿地公园、黔灵山公园、青岩古镇、南江大峡谷、桃源河、南明河沿线街区等一批景区景点，丰富提升餐饮、住宿等服务业态，高端策划宣传，深度开发国内市场，大力拓展境外市场，确保旅游人次和旅游总收入增长30%以上。围绕提升“中国夏季会展名城”品牌影响力，高起点策划、高标准谋划、高水平举办生态文明贵阳国际论坛以及中国科协年会、酒博会、旅交会等重大会展活动，使会展综合经济效益增长30%。筑牢“一个高地”。在继续大力发展传统服务业的同时，积极发展研发设计、科技服务、文化创意、服务外包等高端服务业态，提升省会城市的综合要素配置力，筑牢全省服务业发展高地！

　　三是大力推动信息产业升级。以高新技术产业园、小孟工业园、新天电子元器件基地、数字内容产业园等为基础，打造新型电子元器件、集成电路、LED、新能源电池为主的电子器件产业集群，支持本地智能家电产业加快发展，大力引进电子整机企业，促进民用电子发展和技术水平提升，推动信息产业聚集发展。围绕智慧城市建设，做好信息化顶层设计，统筹整合电子政务、无线城市、三网融合、数字城管、天网工程、智能电网、市民一卡通、社区服务等信息化资源，集中构建云计算信息平台，提升城市智能化、信息化水平。支持获得国家住建部首批智慧城市试点的生态智慧乌当建设。

　　四是大力推动农业产业升级。发挥生态优势和城郊优势，构建高产、优质、高效、生态、安全的现代农业产业体系，提升农业综合效益，拓宽农民增收渠道。提高农业科技水平。进一步抓好农民素质培训、实用技术推广、良种良法普及等重点工作，建设一批设施农业示范基地、标准化菜地良田，提高耕地产出率。加大结构调整力度。围绕都市农业、高效农业、观光农业的发展思路，继续加大粮改经力度，力争粮经比调整到2:8，建设10个现代高效农业示范园区、6个生态农业示范带、100个家庭农场，推进清镇国家现代农业示范区、乌当国家农业科技示范区建设。依托自然优势，大力发展特色生态养殖，力争畜牧业产值占农业总产值的比重提高3个百分点。推进农业产业化经营。扶持壮大“老干妈”、“五福坊”、“贵州龙”等现有农产品加工龙头企业和“黔山牌”、“山花”、“三贵”等品牌产品，新引进和培育龙头企业20家，发展蔬菜、养殖、农机服务等各类农民专业合作组织50个，提高农产品加工转化率3个百分点。

　　（四）更加注重功能完善，提升城镇化发展水平

　　城市是我们共同的家园，城市让生活更加美好。把这座城市建设得更加健康、更加美丽是我们共同的责任！今年，要继续按照“疏老城、建新城”的要求，走集约、智能、绿色、低碳的新型城市化道路，着力提升城市的聚集力、辐射力、带动力和竞争力。

　　一是进一步优化空间布局。按照“世界眼光、国内一流、贵阳特色”的要求，围绕黔中经济区核心区和贵安新区规划布局，以50年至100年不落后的标准，高起点推进“三区五城五带”建设，不断拓展城市发展空间；高质量推进二环四路城市带15个功能板块建设，全力打造功能完善、环境怡人、特色鲜明的生态文明城市精品示范带，构建优化主城与拓展新区的联动圈，提升城市产业发展、公共服务、吸纳就业、聚集人口的功能；高水平规划50个、启动实施20个示范性城市综合体，加快推进未来方舟、假日方舟、花果园、中渝第一城、白云俊发城、万科大都会等重点城市综合体建设，打造一批时尚现代、环境优美、功能复合、带动力强的“城中之城”。

　　二是进一步完善基础设施。加快推进市域快速铁路、城市轨道交通、火车北站、贵阳客运西站、龙洞堡机场改扩建（三期）等项目建设，以及贵阳至瓮安、贵阳至遵义、贵阳至惠水、贵阳至黔西高速公路和黔中路、贵阳至龙里、观山湖至扎佐、白云至修文、天河潭大道、北京东路延伸段等城市干道建设。启动建设奥体中心（二期）项目，完成中华路、中山路地下人防主体工程，抓好公交港湾、公共停车位建设，新建和改造提升25处地下人行过街系统，进一步优化城市“微循环”路网和人行道设施。进一步建强城市供水、供气、供电、供热、通信等基础设施，着力提升城市健康运转的综合要素支撑力。

　　三是全面提升管理水平。推进科学化管理。积极发挥12319公共服务平台作用，加强信息技术和标准化手段在城市管理中的运用，建立部门联动、公众参与和社会协同的城市网格化管理机制，提升城市管理科学化、信息化、法制化、规范化水平。推进精细化管理。制定完善湿地公园、户外广告设置等专项管理办法，突出抓好重要道路沿线、城市出入口、施工工地、公交、公务车辆以及背街小巷、集市、门头等方面的综合整治，建立日常巡查和长效管理机制，让城市“面子”大气美观、“里子”整洁有序。推进人文化管理。坚持“严格执法、文明执法、亲情执法、全心服务”的管理理念，推动生产方式、生活方式和思维方式的变革进步，进一步推广应用“三创一办”的好经验、好做法，广泛深入开展“绿丝带”志愿服务和群众性文明创建活动，提高社会协同和公众参与程度，更好地形成美丽城市·共建共享、幸福家园·共创共享的良好氛围！

　　四是推进城乡一体化发展。按照“区县同城，一体发展”的发展思路，大力加强县城、重点集镇和工业集中区建设，高标准打造23个交通枢纽型、旅游景观型、绿色产业型、工矿园区型、商贸集散型和移民安置型小城镇。加快学校、医院、公共交通、文化设施、商业服务等基本公共服务设施向乡村延伸，着力推动农村公路硬化、标准化农田建设、农村水利及人饮安全、农村危房改造、环境治理等五大工程建设，切实改善农村生产生活条件，提升环境质量，不断让农民与城市居民享受同城化待遇。新建农村公路200公里，新增标准化农田3万亩，完成新一轮农村危房改造5000户，农村污水处理、垃圾转运设施覆盖面提高30%。

　　（五）更加注重改革创新，增强发展活力

　　改革创新是我们加速发展的动力源泉！必须坚持向改革要红利、向开放要效益、向创新要动力，切实做到改革不停顿、开放不止步、创新无止境，同步释放内外潜力。

　　一是继续深化重点领域和关键环节改革。推进行政体制和审批制度改革。稳步推进事业单位分类改革，健全部门职责体系；积极推进符合条件的县（市）改区、乡改镇、乡镇改社区工作。加大政务服务机构标准化建设，实现三级政务服务中心的网上联结；进一步优化土地、矿产资源开发利用审批制度，提高政府对资源的统筹力和调控经济发展的能力。推进财税体制改革。深入推进财政预算管理改革，建立和完善公共财政预算、政府性基金预算、国有资本经营预算和社会保障基金预算有机衔接的预算体系，完善财政支付制度；推行财政投资绩效评价，构建绩效导向型公共财政；探索政府购买公共服务机制，推进非基本公共服务市场化改革；按照国家统一部署，积极推进“营改增”工作。推进投融资体制改革。在严控资金风险的前提下，充分发挥财政性存款的放大作用，鼓励支持各类银行、证券、保险机构、资产管理公司做大做强，拓展金融服务领域，规范市级投融资公司发债和申贷审批制度，全面提高企业、政府融资能力，扩大融资规模，强化融资风险防范。深化国有企业改革。加强和改善经营性国有资产监管，稳步推进投融资公司及实体企业的重组整合。全面启动未改制国有企业改革改制；进一步加大对已改制国有企业的扶持，帮助做大做强做优。推进城乡综合配套改革。推进经开区、乌当区省级新型工业化综合配套改革试点和花溪区、开阳县等4个统筹城乡综合配套改革试点工作。完成农村土地经营权和集体土地所有权确权登记颁证，加快推进农村集体建设用地使用权、宅基地使用权等确权登记工作。在依法自愿有偿的前提下，鼓励农民以多种形式流转土地承包经营权，发展适度规模经营。推进重大项目决策机制改革。健全完善稳定、生态、资金、廉政四大风险评估机制，提高项目投资效益；进一步缩短土地、环评、规划等审批时限，加快项目落地进度；建立企业诚信档案，对诚信企业投资项目简化程序，特事特办。

　　二是积极推进科技创新。实施创新驱动战略，支持创新要素、创新资源向产业、园区、企业集聚，完善产业技术开发、产权交易保护、科技金融服务等平台，加快构建以市场为导向、企业为主体、产学研相结合的区域创新体系，推动科技成果商品化、资本化、产业化。重点实施“5个100科技计划”：鼓励企业自主创新，扶持发展100家创新能力突出、具有自主知识产权的创新型企业、知识产权示范企业；组建100个工程技术中心、重点实验室和产业技术创新战略联盟；实施100个重点科技攻关项目；实施100个重点技术改造项目；培养、引进100个高层次人才。启动建设中科院贵州现代资源技术研究成果转化中心。力争全社会研发经费占生产总值比重达1.8%，科技进步对经济增长贡献率达54.5%，高新技术产业增加值增长30%以上。

　　三是大力提升对内对外开放水平。切实加大招商引资力度。进一步优化招商引资环境，瞄准国内外优强企业，创新“市级统筹、区县负责、园区为主、跨年考核”工作机制，大力推进产业招商、园区招商，积极引进一批关联度大、牵引力强、产业链长的龙头项目，提高资金到位率和项目开工率、投产达产率，确保引进省外到位资金1560亿元以上。提升经济发展外向度。积极推进综合保税区、无水港等开放平台申建工作。深入落实《关于加快开放型经济发展的实施意见》，不断提高外向型经济发展水平，实现外贸进出口总额54亿美元，实际利用外资6亿美元。不断激发经济发展活力。严格落实注册登记、财政税收、融资担保、土地供应、行政规费减免等方面的扶持政策，做到思想上放开、政策上放宽、工作上放手、发展上放活，大力发展实体经济，确保实现民营经济三年倍增目标，使全市民营经济比重超过50%，继续实施“3个20万”扶持政策，新增微型企业5000户，进一步优化经济发展结构，夯实经济发展基础。

　　（六）更加注重以改善民生为重点的社会事业，建设幸福贵阳

　　改善民生是我们的最大责任。要积极顺应群众期待、回应百姓关切，以开展全面小康创建活动为重要载体，按照“三个不能代替”的要求，紧盯“三项核心指标”，严格“三把尺子丈量”，加快在全省率先建成一个“不含水分、百姓满意”的全面小康社会。

　　一是大力提升市民幸福指数。从群众最关心、最直接、最现实的利益问题出发，集中力量办好今年的“十件实事”（详见附件），着力实施“十大民生工程”。实施生态移民工程。着眼于改善群众生活质量、有效保护生态，结合城镇化推进，对生态脆弱区、水源保护区实施生态移民，年内搬迁1086户、4016人。实施充分就业工程。把创造就业岗位作为政府工作的优先目标，经济发展的优先战略，进一步完善创业带动、政策帮扶、城乡统筹三位一体的就业服务体系，做好大中专毕业生、城镇就业困难人员、农村转移劳动力等重点人群的就业服务。实现充分就业社区创建达65%，力争农村“零转移就业家庭”动态为零，城镇新增就业20万人。实施农村扶贫巩固提升工程。按照“三位一体”扶贫格局，夯实产业扶贫基础，促进群众稳定增收；进一步完善贫困人口动态监测和进退管理机制，切实保障好返贫人口以及低保户（五保户）等特殊困难群体的生产生活。五保户集中供养率提高20%以上。实施居民增收工程。大力发展中小微企业和城市服务业，努力促进充分就业，将200万元以下投资规模的农村基础设施项目通过“一事一议”由农民自行组织实施。适度提高城市低保对象补助标准，推动建立用工工资稳定增长和权益保护机制，多渠道促进城乡居民工资性、财产性、经营性、转移性收入增长，确保居民收入与经济增长同步，共享改革发展成果。实施城乡社保体系建设工程。按照“广覆盖、保基本、多层次、可持续”的原则，统筹抓好养老、医疗、失业、工伤、生育保险等城乡社会保障体系建设，实现应保尽保。实施劳动力素质提升工程。组织农村劳动力技能培训1.8万人以上，中高职招生3.3万人以上，培养技能人才3.7万人以上。实施住房保障工程。按照 “政府让利、企业微利、群众得利”的原则，加快棚户区改造和保障性住房建设，让2000户以上住房困难群众搬住新居。实施物价稳控工程。深化价格及价调机制改革，全面推进“平价体系”建设，加大重要农副产品保供基地和平价商店建设，优化布局，提高覆盖面，夯实物价稳控基础。完善社会救助和保障标准与物价上涨挂钩联动机制，稳步提升群众基本生活水平。实施文化惠民工程。支持文化产业发展，创作一批文艺精品，加强新闻媒体、文艺社团的现代传播能力建设；着力打造筑城群众文化品牌，广泛开展送文化进社区、进学校、进农村、进企业活动；抓好文物古迹、非物质文化遗产等保护，以活动为载体把孔学堂打造成为弘扬传统文化基地，积极创建国家公共文化服务示范区。实施幸福家园建设工程。整合资金4亿元，共创共建“新型社区·温馨家园”和“美丽乡村·四在农家”。改造提升社区、村级活动场所，为城乡居民学习培训、自治管理、综合服务、文化活动提供便利条件。

　　二是大力提升群众满意指数。着力优化公共资源配置，推进公共服务均等化，促进社会和谐进步。大力发展教育事业。坚持教育优先发展和优质发展“双优”战略，全年压缩行政开支8%用于教育发展，确保教育投入不低于全市生产总值的4%。完成学前教育三年行动计划，实现乡镇公办幼儿园全覆盖。切实做好“控辍保学”工作，实施“9+3”义务教育及三年免费中等职业教育计划，加强农村中小学寄宿制学校建设，推进义务教育均衡发展；加快实验三中、贵阳六中改扩建和百花湖国际学校建设，优化教育资源布局，提高高中普及率；加快清镇职教城建设，让更多适龄青年接受职业教育培训。鼓励、扶持和规范民办教育，实现各级各类教育健康协调发展。深入开展师德师风教育和教师培训计划，提升教育教学水平。做好新形势下干部教育培训，加快推进市委党校（行政学院）改扩建工作。积极发展卫生事业。组建公共卫生救治中心，开工建设市儿童医院、市残疾儿童康复中心，加快市一医一分院、市二医分院、市疾控中心、市五医等项目建设，加快城市社区卫生服务体系和农村三级卫生服务网络建设，大力推进公立医院机构改革和全科医生执业方式、服务模式改革，实施医疗从业人员在岗培训计划，努力为群众提供安全、优质、方便、价廉的医疗卫生服务。扎实推进社会管理创新。切实巩固城市基层管理体制改革成果，加快社区立法工作，进一步理顺社区管理和服务职能，实现网格化、规范化管理。创新信访工作机制，有效解决群众合理诉求。充分发挥新社会组织、新经济组织和民间社团组织的积极作用，进一步加强管理和服务，使之成为推进社会文明、促进经济发展、构建和谐社会的重要力量。认真开展第三次经济普查工作，继续推进双拥优抚、民族宗教、外事侨务、对台、移民、史志、档案、老龄、残疾人、司法行政、国家安全等事业发展，支持国防建设和法院、检察院工作，支持工青妇、关工委、计生协会等组织做好工作。

　　三是大力提升社会安全指数。加强社会治安综合治理，强化政法队伍和执法装备建设，建立系统性、协同性、立体化社会治安防控体系，依法严厉打击危害群众生命财产安全的一切犯罪活动，着力增强群众安全感。健全突发公共事件预警和应急处置机制，提高应对突发事件的能力。推进贵阳率先基本实现气象现代化，提高气象灾害防御能力。扎实抓好安全生产、食品药品安全等工作，促进社会和谐稳定。

　　三、切实加强政府自身建设

　　新形势新任务对政府工作提出了新要求，我们将进一步加强政府自身建设，建设一支勤政、廉洁、务实、高效的公务员队伍，切实做到干部清正、政府清廉、政治清明，为科学发展、后发赶超、提前小康提供有力保障。

　　（一）切实加强作风建设，增强执行力

　　按照中央“八项规定”、省委“十项规定”、“十破十立”和市委“敢、实、清”的要求，切实改进会风、文风、作风，进一步增强求真务实、精益求精、扎实勤勉的意识，坚决反对形式主义、官僚主义，用更多精力广泛深入基层、联系群众、掌握实情、解决问题、推动发展，以全新的思维方式和务实的工作作风，增强执行力。

　　（二）切实加强制度建设，增强发展力

　　加强重大事项决策、绩效管理、政务公开等制度建设，严格依照法定权限和程序行使权力、履行职责，依法接受人大及其常委会的法律监督和工作监督，自觉接受政协的民主监督，主动接受司法监督、舆论监督和社会监督，切实强化审计监督，形成严格规范的制度体系和监督体系，确保行政权力在阳光下运行，行政决策在制度中推进，以严格的守法意识、规范的监督机制、高效的行政制度，增强发展力。

　　（三）切实加强效能建设，增强服务力

　　把务实高效作为一种追求、一种品质和一种责任，在服务企业、服务群众、服务基层中转变政府职能，严格控制机构编制，降低行政成本，进一步优化审批环节、缩短审批时间、提高办事效率，努力营造公平透明的政策环境、优质高效的服务环境、安全稳定的法制环境、竞争有序的市场环境、健康诚信的人文环境。全面落实首问负责制、限时办结制、责任追究制，从严查处办事拖拉、推诿扯皮、吃拿卡要、敷衍塞责等不作为、慢作为、乱作为的不正之风，从严治理“门难进、脸难看、话难听、事难办”的衙门邪气，以行风大转变、效能大提升、环境大改善，增强服务力。

　　（四）切实加强廉政建设，增强公信力

　　认真贯彻落实中纪委二次全会精神，严格执行领导干部廉洁从政各项规定，着力完善惩治和预防腐败体系，“把权力关进制度的笼子里，形成不敢腐的惩戒机制、不能腐的防范机制、不易腐的保障机制”；加大查办违纪违法案件的力度，坚持“老虎”、“苍蝇”一起打，严肃查办滥用职权、玩忽职守、贪污贿赂、腐化堕落案件，严肃查办发生在重点领域和关键环节的腐败案件，严肃查办商业贿赂案件，保持惩治腐败的高压态势；加大纠风和专项治理力度，切实解决损害群众利益的突出问题，确保人民赋予的权力真正为人民谋利益，厉行勤俭节约、制止奢侈浪费，以公正清廉、实干为民的新风尚、新气象，增强公信力。

　　各位代表，把贵阳建成全国生态文明示范城市是我们共同的追求，全面建成小康社会是我们共同的期待。让我们紧密团结在以习近平同志为总书记的党中央周围，以党的十八大精神为指引，在省委、省政府和市委的坚强领导下，弘扬“知行合一，协力争先”的贵阳精神，以更加开阔的视野，更加进取的精神，更加扎实的作风，更加优异的成绩，阔步迈向生态文明新时代！
